# Integrated Metabolomics and Network Pharmacology Study on Immunoregulation Mechanisms of* Panax ginseng* through Macrophages

**DOI:** 10.1155/2019/3630260

**Published:** 2019-06-25

**Authors:** Junjie Hao, Huangwanyin Hu, Jing Liu, Xuan Wang, Xiaoyi Liu, Jiabo Wang, Ming Niu, Yanling Zhao, Xiaohe Xiao

**Affiliations:** ^1^College of Pharmacy, Chengdu University of Traditional Chinese Medicine, Chengdu 611137, China; ^2^Department of China Military Institute of Chinese Materia, the Fifth Medical Center, Chinese PLA General Hospital, Beijing 100039, China; ^3^Chengde Medical University, Chengde 067000, China; ^4^School of Traditional Chinese Medicine, Capital Medical University, Beijing 100039, China

## Abstract

*Panax ginseng* (PG) is a widely used functional food and herbal with immunoregulation activity. Currently, immunoregulation studies of PG mainly focused on the specific actions of individual constituents. However, the integral immunoregulation mechanisms of PG need further research. In this study, an integrated metabolomics and network pharmacology approach were used to investigate it. High-content screening was used to evaluate macrophage phagocytosis activity of PG. Untargeted metabolomics profiling of murine macrophage cells with UHPLC-Q-TOF-MS and a multivariate data method were performed to discover the potential biomarkers and metabolic pathways. Then, a macrophage phenotype related “ingredients-targets-metabolites” network of PG was constructed using network pharmacology for further research. As a result, PG can significantly enhance macrophage phagocytosis of GFP-*E. coli*. A total of twenty potential biomarkers and ten main pathways for which levels changed markedly upon treatment were identified, including glycerophospholipid metabolism, glutathione metabolism, choline metabolism, and taurine metabolism. Twenty compounds of PG associated with metabolomic changes were selected by the network pharmacology analysis, including ginsenoside Re, ginsenoside Rg1, frutinone A, and kaempferol. The network pharmacology results also showed that PG can polarize macrophages to both M1 and M2 phenotype but may be prone to M2 phenotype. In conclusion, our results indicated that PG may be prone to polarize macrophages to M2 phenotype by mainly regulating the glutathione and choline metabolism, which was related to twenty compounds of PG.

## 1. Introduction

*Panax ginseng* (PG) is a type of health food used around the world and is also a widely used herbal in Asian countries [1]. PG refers to the dry root and rhizome of* Panax ginseng* C.A. Meyer (Araliaceae) and is often extracted by water and alcohol. PG and its major pharmacologically active ingredients, ginsenosides, have various pharmacological properties, such as regulating central nervous system, immunomodulation, anti-inflammation, anticancer, antidiabetes, antioxidative, antifatigue [2–5]. PG is an immune modulator and exhibits effects on maintaining immune homeostasis and enhancing resistance to illness or microbial attacks [6]. It has been proved that PG extract and components have immune-regulatory properties through modulation of macrophages, which are the most versatile cells of human immune system to form the first line of defense against pathogens [7–9]. For example, the polysaccharide fraction of PG has immunopotentiating effects on macrophages [10]. Oligopeptides can regulate innate and adaptive immune responses in mice via increased macrophage phagocytosis capacity [11]. Ginsenoside Rg1 can regulate innate immune responses in macrophages through differentially modulating NF-*κ*B and PI3K/Akt/mTOR pathways [12]. However, most of these studies in the immunoregulation of PG have focused on the specific actions of individual constituents. It is not comprehensive enough to investigate the integral immunoregulation mechanisms of PG, which has multiple components and multiple targets [13]. Therefore, it is necessary to research the integral immunoregulation mechanisms of PG with systems biology approach.

Metabolomics, which is the profiling of metabolites in biofluids, cells and tissues, is a powerful approach to insight into the global changes in biological systems. Hence, metabolomics represents an excellent developing prospect in revealing the action mechanism of herb medicines [14]. However, the disturbances of metabolic pathways by the metabolomics analyses are still far from fully clarifying how the herbs work. Network pharmacology has been commonly used in recent years to research and predict the interaction mechanism between a drug and its targets in the context of biological networks and interconnected pathway. It gives a systems-level of understanding the interaction between disease features, bioactive agents, and drug targets [15–17]. Recently, network pharmacology, as a holistic and efficient technique to study the role of herbs, can help to interpret the underlying mechanisms of herbs by understanding the active compounds and therapeutic targets of them [18], which can provide a better understanding of the complex relationship between PG ingredients and subnetworks. Moreover, the integrated metabolomics and network pharmacology strategy has been successfully used to explore the interactions between organisms and herbs, which brings great inspiration to elucidate the immunoregulation mechanisms of PG through macrophages [19].

In this study, the possible immunoregulatory mechanisms of PG were investigated by integrating nontargeted cell metabolomics and network pharmacology. The immunoregulation of PG was assessed through the phagocytosis activity of macrophage cells engulfing GFP-*E. coli *(Escherichia coli expressing a green fluorescent protein) by high-content screening (HCS), which combines the efficiency of high-throughput techniques with the ability of cellular imaging [20, 21]. Furthermore, cell metabolomics was used to screen the potential biomarkers related to the action mechanisms of PG [22, 23], and an “ingredients-targets-metabolites” network was constructed by network pharmacology approaches to clarify the main immunoregulation targets and related components [24]. The result showed that 20 compounds of PG may contribute to enhance the phagocytosis activity of macrophages by regulating the metabolic changes in 10 main pathways, such as glycerophospholipid metabolism, glutathione metabolism, taurine metabolism. In addition, the network pharmacology results also showed that there were eight M1 macrophage proteins and thirty-seven M2 macrophage proteins associated with potential biomarkers, including peroxisome proliferator activated receptor gamma, interleukin 4, interleukin 6, tumor necrosis factor, nitric oxide synthase 2, and toll like receptor 7. The results demonstrated that PG can polarize macrophages to both M1 and M2 phenotype but may be more prone to M2 phenotype.

## 2. Materials and Methods

### 2.1. Chemicals and Reagents

Foetal bovine serum (FBS) and Dulbecco's modified Eagle's medium (DMEM) were purchased from Biowest (Nuaillé, France) and HyClone (Logan, Utah, USA), respectively. Escherichia coli BL21 (DE3) pLys chemically competent cells and the plasmid pGFPuv (EX-MCHR-B01) were purchased from TransGen Biotech (Beijing, China) and GeneCopoeia (Guangzhou, China), respectively. Acetonitrile and methanol (HPLC grade) were purchased from Merck (Darmstadt, Germany) and Burdick and Jackson (Ulsan, Korea), respectively. Double-distilled water was purified using a Millipore water purification system (Millipore, Bedford, MA). Other chemicals were of analytical grade. Five-year-old* Panax ginseng* was provided by China Medico Corporation (China). Ginsenosides Re, Rg1, Rb1, Rf, Ro, and Rd were purchased from Chengdu Pufei De Biotech Co. Ltd. (Chengdu, China).

### 2.2. Extract Preparation of PG and UPLC-PDA Conditions

Ten grams of powdered sample were extracted in a 250 ml Erlenmeyer flask for 1 h at 100°C by refluxing with 100 mL of 70% (v/v) ethyl alcohol twice and then lyophilized. The lyophilized powder of the extract was approximately 26.13% of the original weight and was dissolved prior to use.

The prepared samples were analyzed using a Waters ACQUITY UPLC system (Waters, Milford., MA, USA) coupled with a photodiode array detector (PDA). A Waters ACQUITY UPLC BEH C18 column (2.1 × 50 mm, 1.7 *μ*m) was employed to achieve separation at 35°C. The mobile phases were water (solvent A) and acetonitrile (solvent B) with gradient elution as follows: 0–8.0 min, 10%–19% B; 8.0–13.0 min, 19%–21% B; 13.0–21.0 min, 21%–32% B; 21.0–29.0 min, 32%–32%; 29.0–35.0 min, 32%–50%. Elution was performed at a flow rate of 0.35 mL/min and UV measurements were obtained at 203 nm.

### 2.3. GFP-E. coli, Cell and Animal

A green fluorescent protein-expressing Escherichia coli (*GFP-E. coli*) containing an additional ampicillin resistance gene to allow antibiotic selection was transformed according to the specifications required. GFP-*E. coli* was cultured in Luria Bertani broth (Aobox Biotechnology, Beijing, China) in 37°C. One hundred *μ*g/mL ampicillin (Amresco, Houston, Texas, USA) was used for antibiotic selection of bacteria containing GFP expression plasmids and for preventing other bacterial contamination. Bacteria density was determined by measurement of OD600, and OD600 of 1.00 was approximately 3.0e8 CFU /ml [25].

Murine macrophage RAW 264.7 cells were purchased from Institute of Basic Medicine, Chinese Academy of Medical Sciences (Beijing, China) and maintained in complete DMEM supplemented with 10% heat-inactivated FBS, 1% penicillin-streptomycin (Macgene, Beijing, China) in a humidified incubator at 37°C at an atmosphere of 5% CO_2_.

The animal study was approved by the Institutional Animal Care and Use Committee of 302 Military Hospital (Approval ID: IACUC-2018-015). Male BALB/c mice weighing 20 ± 2 g were purchased from Vital River Laboratory Animal Technology Co., Ltd. (Beijing, China) and the room temperature was regulated at 25 ± 2°C with 50 ± 5% humidity. A 12-h light/dark cycle was set, and the animals were provided free access to a standard diet and water.

### 2.4. High-Content Screening Analyses of Phagocytosis Activity

The experiment with murine macrophage RAW 264.7 cells was divided into several groups: control group, PG-treated groups (115 *μ* g/mL, 230 *μ* g/mL, 345 *μ* g/mL, 460 *μ* g/mL, 920 *μ* g/mL and 1840 *μ* g/mL), and LPS (150 ng/mL) group. As macrophages activated by LPS were more effectively engulf substances, e.g., bacteria, LPS was used as positive control agent [26]. Cells were diluted into a final concentration of 3 × 10^5^ cells/mL, and 100 *μ*L of prewarmed growth medium were added to each well using a multidrop-96 (Thermo Scientific, Massachusetts, USA). After 16 h of incubation, the medium was replaced with an extract of various concentrations of PG and LPS for 12 hours. Then, the medium was replaced by the medium containing GFP-*E. coli* (100 GFP-*E. coli*/cells), and the sample was incubated for another 2 h. The study on the methodology of phagocytosis activity was shown in Figure S1. Then, cells were fixed with 4% formaldehyde and dyed with DAPI (Roche, Basel, Switzerland). The samples were washed with PBS thrice before detection.

Twelve BALB/c mice were randomly divided into control group and PG-treated group (0.75g/kg) with six mice each. The control groups were orally administered with the same volume of distilled water. All doses were administered by gavage once a day for 7 consecutive days. Peritoneal macrophages were obtained by washing out the peritoneal cavities of mice and removing nonadherent cells. Then the macrophages (3 × 10^6^ cells/mL, 100 *μ*L each well) were added to a multidrop-96. After 4 h of incubation, medium containing GFP-*E. coli* was added and the other condition was the same as the murine macrophage RAW 264.7 cells above.

Image acquisition was performed using the Array Scan High-Content Systems (Thermo Scientific, Massachusetts, USA), and images were analyzed using the Array Scan XTI (the Array Scan software algorithm was used to perform analysis). A 20 X objective lens was used to analyze 16 fields for a total of 81 in every well of the multidrop-96. Cell numbers were counted using software based on DAPI dye staining. Phagocytic percent was calculated to reflect the effect of macrophage phagocytosis [27].

(1) Phagocytic  percent  %=average  phagocytic  index  of  every  groupaverage  phagocytic  index  of  control  group×100%,Average  phagocytic  index=fluorescence  intensity  of  GFP-E.  coli  phagocytosedcell  number.

### 2.5. Untargeted Cell Metabolomics Analyses

*(1) Grouping and Administration. *The cell metabolomics experiment was divided into four groups: control group, PG low-dose group (115*μ*g/L), PG middle-dose group (230 *μ*g/L) and PG high-dose group (345 *μ*g/L). Cells were diluted into a final concentration at 5 × 10^6^ cells/mL, and 2 mL of prewarmed growth medium was added to each well using a multidrop-6 (Corning, New York, USA). After 16 h of incubation, the medium was replaced by new medium containing various concentrations of PG extracts for 12 h. The medium and the cell samples were removed and washed with PBS thrice. Next, the methanol-water (4:1, V/V solution, standing 20 min at 4°C before use) was added. Cell samples were scraped with a cell scraper and placed in cell cryotube (Thermo Scientific, Massachusetts, USA). Then, the cell samples were quenched by liquid nitrogen to stop the enzymatic activity and ruptured by ultrasonic breakers (0°C, power 20%, 3 min, opened 5 S, closed 5 S). Finally, the cell samples were centrifuged at 12,000 rpm for 10 min at 4°C to remove any solid debris and filtered through 0.22-*μ*m membranes before LC/MS analysis.

*(2) Chromatography and Mass Spectrometry Conditions. *The metabolic profiling analysis was performed on an Agilent 6550 iFunnel Q-TOF LC/MS (Agilent Technologies, USA). A ZORBOX 300 SB-C18 analytical column (2.1 mm i.d. × 100 mm, 1.8 *μ*m i.d., Agilent Technologies, USA) was used for separation at a temperature of 30°C with a flow rate of 0.30 mL/min. The injected sample volume was 2 *μ*L and the composition of mobile phase was A (acetonitrile spiked with 0.1% formic acid) and solvent B (water spiked with 0.1% formic acid). The following gradient was used: 100% B over 0-1.0 min, 100–60% B over 1.0–9.0 min, 60–10% B over 9.0–19.0 min, 10–0% B over 19.0–21.0 min, and 100% A over 21.0–25.0 min. 10 *μ*L of each sample were pooled as a quality control (QC) sample to ensure the stability and repeatability of the systems [28].

For mass spectrometry, the Agilent 6550 Q-TOF/MS with an electrospray ionization source (ESI) in both positive and negative mode was used. The electrospray source parameters were fixed as follows: electrospray capillary voltage was 3.5 kV in negative ionization mode and 3.5 kV in positive ionization mode. The mass range was set from 80 to 1000 m/z. Gas temperature was 225°C in negative ionization mode and 225°C in positive ionization mode. Gas flow was 13 L/min. The nebulizer was 20 pisg (negative) and 20 pisg (positive). Sheath gas temperature was 275°C, and sheath gas flow was 12 L/min. Nozzle voltage was 2000 V in both negative and positive modes. For internal mass calibration during the MS analysis, reference masses 121.0509 (Purine, [C5H4N4+ H] +) and 922.0098 (HP-0921, [C18H18O6N3P3F24+H] +) were used in positive mode, and 112.9856 (TFANH4, [C2H4O2NF3− NH4] −) and 1033.9881 TFANH4 + HP-0921, [C20H22O8N4P3F27− NH4] −) were used in negative mode.

*(3) Data Processing and Pattern Recognition Analysis. *All data were preprocessed with Profinder (version B.06.00, Agilent Technologies, USA). Multivariate analysis was performed using principal component analysis (PCA) and orthogonal partial least-squares discriminant analysis (OPLS-DA) in the SIMCA-P+ 13.0 (Umetrics, Umeå, Sweden) software. Variables with a VIP value (VIP ≥ 1.0) and |p(corr)| ≥ 0.5 in the OPLS-DA model were selected as potential biomarkers.

*(4) Metabolites Identification and Metabolic Pathway Analysis. *Metabolites with significant changes were selected as potential biomarkers using fold-changes and p values (fold-change value >1.5 and p value <0.05) calculated by MetaboAnalyst 3.0 software (http://www.MetaboAnalyst.ca/) [29]. Multiple comparisons among groups were performed by one-way analyses of variance (ANOVA). All metabolites were tentatively identified based on the accurate mass charge ratio using the online METLIN database (http://www.metlin.scipps.edu) and HMDB database (http://www.hmdb.ca/). Here, 20 ppm was set as the accepted mass error. Pathway analysis of the identified potential biomarkers was performed with MetaboAnalyst 3.0 (http://www.metaboanalyst.ca) based on the pathway library of Mus musculus (mouse).

### 2.6. Network Pharmacology Analyses

The main chemical components and their targets of PG were obtained from TCMSP (traditional Chinese medicine systems pharmacology database and analysis platform http://lsp.nwu.edu.cn/tcmsp.php) and Pharm Mapper (http://lilab.ecust.edu.cn/pharmmapper/index.php). The enzymes in the control of metabolites were obtained from the HMDB database and KEGG database. Protein-protein interaction network database (DIP, http://dip.doe-mbi.ucla.edu/dip/Main.cgi and IntAct, https://www.ebi.ac.uk/intact/) was used to establish the macrophage phenotype related “ingredients-targets-metabolites” and the interaction proteins were selected with the network depth no more than 2. The network was generated using Cytoscape 3.6.1 software (Cytoscape Consortium, California, USA). The proteins of M1 macrophage and M2 macrophage were obtained from GeneCards (https://www.genecards.org/).

## 3. Results

### 3.1. Chemical Profiling and Component Identification of PG Extract

To ensure the quality of PG, the chemical fingerprint was established by a UPLC system. The same batch PG used for the metabolomics experiment was extracted twice with 70% ethyl alcohol. The UPLC profile of PG extract and the reference mixture were shown in Figure S2, seven main components, including ginsenoside Rg1, ginsenoside Re, ginsenoside Rf, ginsenoside Rb1, ginsenoside Rc, ginsenoside Ro, and ginsenoside Rd, were identified and marked according to their retention time compared with standards.

### 3.2. PG Enhances Macrophage Phagocytosis of GFP-E. Coli

**Figure 1:**
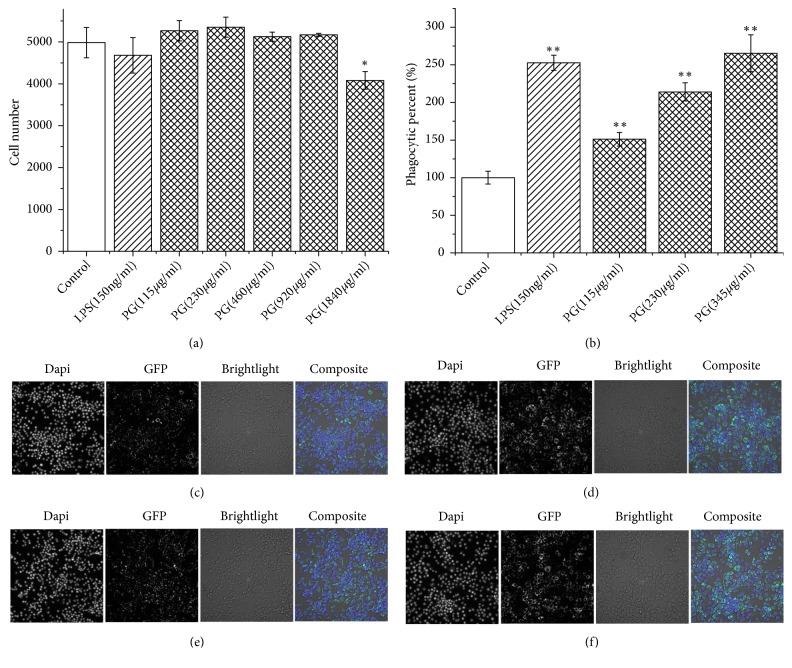
High-content screening analyses of murine macrophage RAW 264.7 using a 20 X objective lens in Multidrop-96: images of 16 fields (a total of 81 fields in every well) were acquired and analyzed. (a) Cell number of control group and PG-treated groups; (b) phagocytic percent % of every group; (c-f) images of 1 field obtained by high-content screening. The “Dapi” panels represent nuclei stained with 4′,6-diamidino-2-phenylindole dihydrochloride. The “GFP” panels represent GFP-*E. coli*. The “composite” panels were merged with three panels above; (c) control group; (d) LPS-treated group; (e) PG-treated group at 115 *μ*g/ml; (f) PG-treated group at 345 *μ*g/ml. ^*∗*^*P* < 0.05, ^*∗∗*^*P* < 0.01, compared with control group.

**Figure 2:**
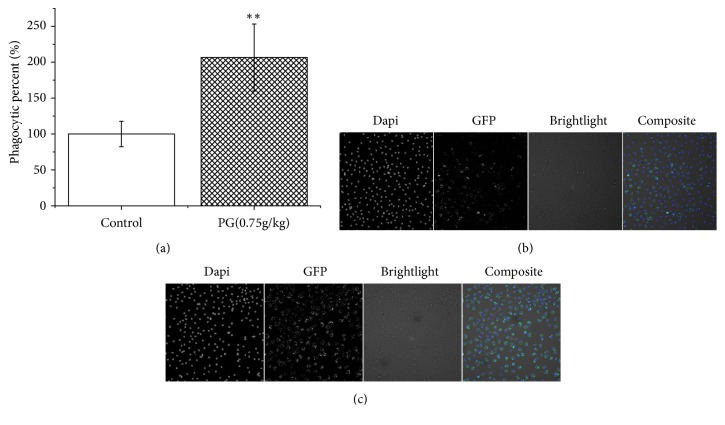
High-content screening analyses of peritoneal macrophages of BALB/c mice. (a) Phagocytic percent % of control group and PG-treated groups; (b) control group; (c) PG-treated group at 0.75g/kg. ^*∗∗*^*P* < 0.01, compared with control group.

Both* in vivo* and* in vitro* experiments were used to investigate the PG's effect on macrophage phagocytosis. Murine macrophage RAW 264.7 was used for the phagocytosis analysis* in vitro*. The macrophage viability with the CCK-8 vital and the cell number calculated by high-content screening were used to estimate the macrophage toxicity of PG and LPS (Figures 1(a) and S3). The results revealed that there was no cytotoxicity in the PG extracts (115-460 *μ*g/ml) and the LPS (150 ng/ml) as no significant difference was found contrasted with control group. In addition, there were promoting effects in PG extracts (115-230 *μ*g/ml) group but without significant difference in cell proliferation of RAW 264.7 (*P* > 0.05) compared to control group. However, PG in the concentration of 1840 *μ*g/ml significantly decreased cell number when compared with control group (*P* < 0.05). The results showed that PG could promote cell proliferation in a certain concentration range, while higher concentration may have cytotoxicity in RAW 264.7. PG extracts at 1000 *μ*g/ml could not significantly decrease cell viability of peripheral blood mononuclear cell (PBMC) [30]. Moreover, no adverse reactions were observed in rats even treated with 2000 mg/kg/day PG extract [31]. Furthermore, macrophage phagocytic percentage (%) was also used to reflect the effect of macrophage phagocytosis. As shown in Figure 1(b), PG can enhance macrophage phagocytosis of GFP-*E. coli in vitro* at the concentration of 115-345 ug/ml. Also, as shown in Figure 2, PG can enhance peritoneal macrophages of BALB/c mice phagocytosis of GFP-*E. coli* by gavage to the PG extracts (0.75g/kg).

### 3.3. Metabolomics Results

**Figure 3:**
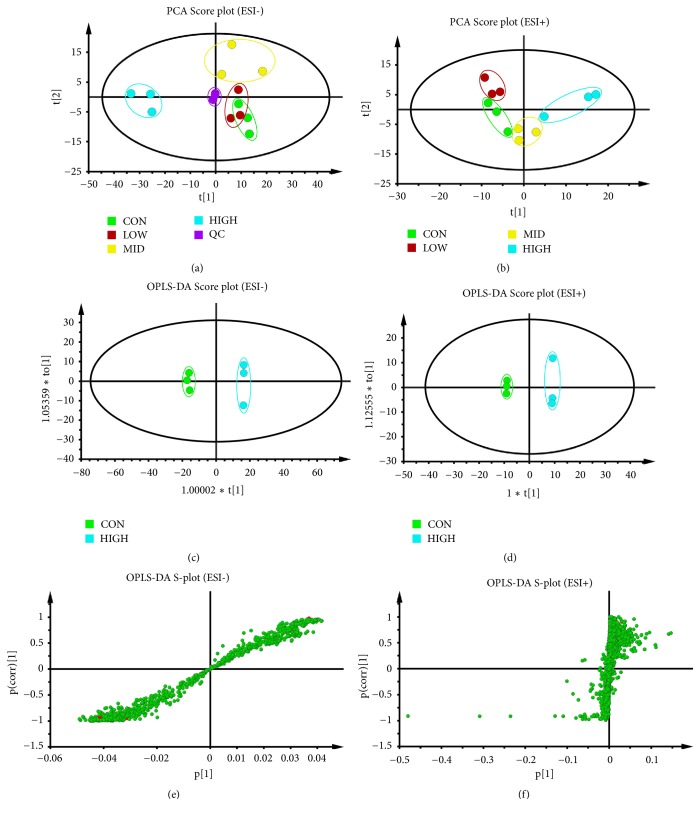
Score plots of control group, groups treated with PG extract in different concentrations, and QC from PCA in the ESI− mode (a) and ESI+ mode (b). QC indicates the quality control group. OPLS-DA analysis of the data derived from the ESI- mode. OPLS-DA score plots (c, d) and S-plot of the OPLS-DA model (e, f) for the pair-wise comparisons between the control and high-dose group of PG. The axes that are plotted in the S-plot from the predictive component are p1 vs. p(corr)1, representing the magnitude (modelled covariation) and reliability (modelled correlation), respectively. The points in red indicated the identified potential biomarkers.

*(1) PCA and OPLS-DA Analysis. *An unsupervised PCA statistical method was used to study the cell metabolic differences between the control group and different concentrations groups of PG extract. The score plots of PCA analysis of ESI- mode and ESI+ mode was presented in Figures 3(a) and 3(b), respectively. As shown in Figure 3(a), a quality control sample (QC, mixing 100 *μ*L of each sample) clustered closely in both PCA score plots, demonstrating the stability of the LC/MS system throughout the entire analysis. In addition, an obvious separation trend is observed between the control group and groups with different concentrations of PG extract in both PCA models, indicating a considerable metabolite difference among these groups.

The high-dose treatment group of PG (345 *μ*g/ml) exhibited the best phagocytosis-promoting effect. Thus, OPLS-DA was used to investigate significant differences of macrophages metabolites between control group and high-dose group of PG to identify potential biomarkers. Figures 3(c)–3(f) display the result of the OPLS-DA model derived from ESI- and ESI+ analyses. The score plots (Figures 3(c) and 3(d)) exhibited good model fitness, and the high-dose treatment group can be separated from control group very clearly using data from the ESI- and ESI+ analyses. The OPLS-DA model demonstrated good predictive ability with a R2Y (cum) of 0.83 and Q2 (cum) of 0.985 in ESI- model and a R2Y (cum) of 0.907 and Q2 (cum) of 0.988 in ESI+ model, Table S2.

**Figure 4:**
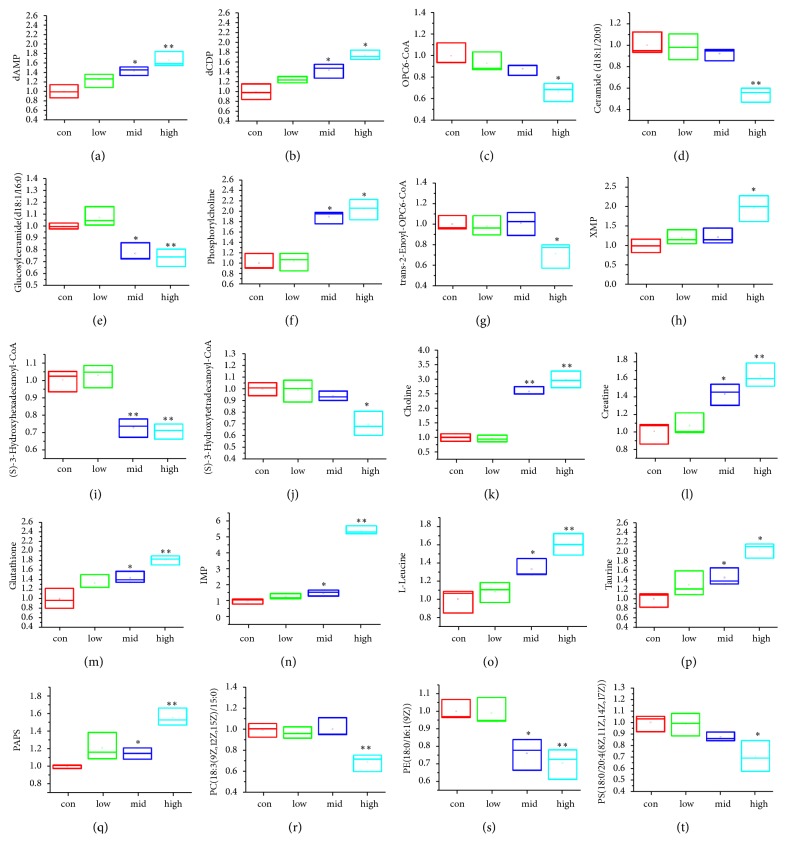
Variations in the trends of the cell metabolites that are potential biomarkers in both the control group and PG-treated group. The Y-axis represents the fold of each group/control group. The X-axis indicates the control group, PG low-dose group (115 *μ*g/L), PG middle-dose group (230 *μ*g/L), and PG high-dose group (345 *μ*g/L). (a–t) are the variations in the trends of Deoxyadenosine monophosphate (dAMP), 2′-Deoxycytidine diphosphate (dCDP), OPC6-CoA, Ceramide (d18:1/20:0), Glucosylceramide (d18:1/16:0), Phosphorylcholine, trans-2-Enoyl-OPC6-CoA, Xanthylic acid (XMP), (S)-3-Hydroxyhexadecanoyl-CoA, (S)-3-Hydroxytetradecanoyl-CoA, Choline, Creatine, Glutathione, Inosine 5′-monophosphate (IMP), Leucine, Taurine, 3′-Phosphoadenylyl sulfate (PAPS), PC(18:3(9Z,12Z,15Z)/15:0), PE(18:0/16:1(9Z)), and PS(18:0/20:4(8Z,11Z,14Z,17Z)). ^*∗*^*P* < 0.05, ^*∗∗*^*P* < 0.01, compared with control group.

*(2) Metabolites Identification and Metabolic Pathway Analysis. *The potential biomarkers were selected by comparing the discrimination of the macrophage metabolites between the control group and high-dose group of PG with VIP > 1, |p(corr)| ≥ 0.5,* P* < 0.05 and |Fold| ≥ 1.5. The metabolites in the ESI+ and ESI− mode analyses were combined and identified with the accurate mass charge ratio using the online METLIN database. Twenty metabolites were identified and selected as the potential biomarkers and summarized in Tables 1 and S1 with their corresponding retention time, mass, formula, and variation trends. To further illustrate the metabolic changes among the control group and PG-treated groups, the relative area intensities of the potential biomarkers were plotted in Figure 4. It shows that the concentrations of Deoxyadenosine monophosphate (dAMP), Leucine, Creatine, 3′-Phosphoadenylyl sulfate (PAPS), 2′-Deoxycytidine diphosphate (dCDP), Glutathione, Choline, Phosphorylcholine, Xanthylic acid (XMP), Taurine, and Inosine 5′-monophosphate (IMP) were significantly increased, whereas the levels of Ceramide (d18:1/20:0), PC(18:3(9Z,12Z,15Z)/15:0), (S)-3-hydroxytetradecanoyl-CoA, PS(18:0/20:4(8Z,11Z,14Z,17Z)), (S)-3-hydroxyhexadecanoyl-CoA, PE(18:0/16:1(9Z)), trans-2-Enoyl-OPC6-CoA, Glucosylceramide (d18:1/16:0), and OPC6-CoA were significantly decreased in the PG-treated group compared with the control group.

**Figure 5:**
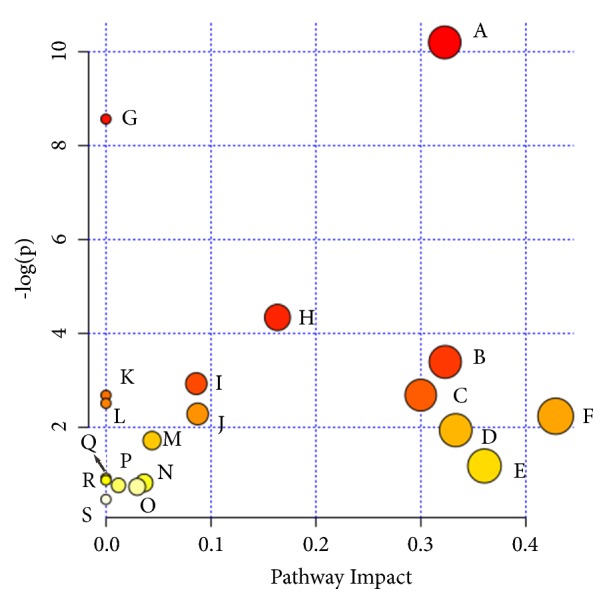
Summary of pathway analysis with MetaboAnalyst 3.0. (A) Glycerophospholipid metabolism; (B) sphingolipid metabolism; (C) sulfur metabolism; (D) valine, leucine, and isoleucine biosynthesis; (E) glutathione metabolism; (F) taurine and hypotaurine metabolism; (G) alpha-Linolenic acid metabolism; (H) purine metabolism; (I) fatty acid elongation in mitochondria; (J) fatty acid metabolism; (K) glycine, serine and threonine metabolism; (L) linoleic acid metabolism; (M) glycosylphosphatidylinositol (GPI)-anchor biosynthesis; (N) pyrimidine metabolism; (O) primary bile acid biosynthesis; (P) arginine and proline metabolism; (Q) arachidonic acid metabolism; (R) valine, leucine, and isoleucine degradation; and (S) aminoacyl-tRNA biosynthesis.

MetaboAnalyst was used to perform the pathway enrichment analysis against the potential biomarkers. Alterations in the potential biomarkers suggested that PG extraction can regulate lipid metabolism, amino acid metabolism, metabolism of other amino acids, nucleotide metabolism and so on through macrophage. The most enriched pathways, including alpha-Linolenic acid metabolism; glycerophospholipid metabolism; sphingolipid metabolism; fatty acid degradation; glutathione metabolism; taurine and hypotaurine metabolism; glycine, serine, and threonine metabolism; valine, leucine, and isoleucine biosynthesis; purine metabolism; and pyrimidine metabolism, are shown in Figure 5 and Table S3.

### 3.4. Network Pharmacology Results

**Figure 6:**
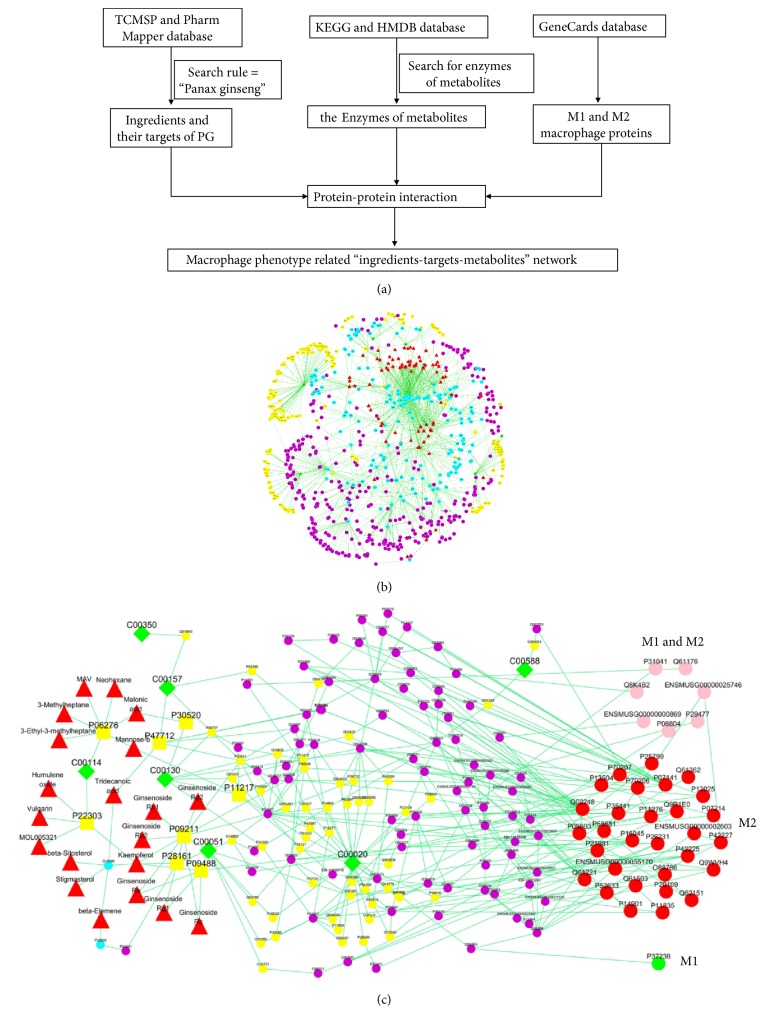
Flow chart of network pharmacology analysis (a), the “ingredients-targets-metabolites” network of PG (b), and the macrophage phenotype related “ingredients-targets-metabolites” network (c). The red triangles represent the active chemical constituents of PG. The blue dots represent the targets for PG constituents. The green diamonds represent the metabolites. The yellow dots represent the metabolic proteins and the yellow squares represent the common proteins of PG constituents and metabolites. In figure (b), the pink dots represent common proteins in M1 and M2 macrophages. The red dots represent independent proteins in M1 macrophages and the green dots represent independent proteins in M2 macrophages.

To further illustrate the potential mechanism of PG on the macrophage phagocytosis, an “ingredients-targets-metabolites” network was constructed and analyzed by a network pharmacology approach. As shown in Figure 6, there are 82 PG's ingredients, 193 drug targets, and 195 metabolic proteins (the enzymes in the control of metabolites) included by database searching and literature mining. Based on the analysis of the network, twenty ingredients associated with seven metabolites were selected, including ginsenoside Rb2, ginsenoside Re, ginsenoside Rc, ginsenoside Rg1, ginsenoside Rg2, ginsenoside Rb1, frutinone A, humulene oxide, kaempferol, and vulgarin. The ADME indexes of these ingredients from TCMSP database including OB (oral bioavailability), Caco-2 (cell permeability), and DL (drug-likeness), based on chemometric method for the prediction in silico models, are listed in Table S4 [32]. The detail interactions of ingredients and metabolites are presented in Figure 6(b) and Tables S4 and S5. These twenty ingredients may be the key components of PG in regulating macrophage activation in the cell-mediated immunity.

Macrophage functional heterogeneity is defined by two activation states, classically activated macrophage (M1) and alternatively activated macrophage (M2). M1 macrophages are characterized by a proinflammatory phenotype and tumoricidal activity, while M2 macrophages contribute more to the regulatory functions in tissue repairment. A total of 32 M1 macrophage proteins and 106 M2 macrophage proteins were obtained from GeneCards database. Among them, there were 27 common macrophage proteins in both M1 and M2 macrophages, 5 independent proteins in M1 macrophage and 89 independent proteins in M2 macrophage, respectively. Next, network pharmacology analysis was performed for further analysis. As shown in Figure 6(c) and Table S6, 8 M1 macrophage proteins and 37 M2 macrophage proteins were associated with potential biomarkers. Among them, there were 7 common proteins in M1 and M2 macrophage (interleukin 4, interleukin 6, tumor necrosis factor, nitric oxide synthase 2, etc.), 1 independent proteins in M1 (peroxisome proliferator activated receptor gamma), and 30 independent protein in M2 macrophages (toll like receptor 7, interleukin 13, nuclear factor kappa B subunit 1, etc.), respectively. PG can polarize macrophages to both M1 and M2 phenotype but may be prone to M2 phenotype to enhance macrophage phagocytosis and its immunoregulation function.

Furthermore, KEGG and Gene Ontology (GO) pathway analysis of the common proteins of PG constituents and metabolites were performed using DAVID database (https://david.ncifcrf.gov/). As shown in Table 2, KEGG pathways were primarily related to glutathione metabolism and glycerophospholipid metabolism. Notably, GO enrichment analysis results indicated that molecular function was mainly related to glutathione binding, glutathione transferase activity, acetylcholinesterase activity, cholinesterase activity, and so on. The major biological processes were glutathione metabolic, glutathione derivative biosynthetic, and linoleic acid metabolic. The main cellular components were cytosol and cytoplasm.

## 4. Discussion and Conclusion

**Figure 7:**
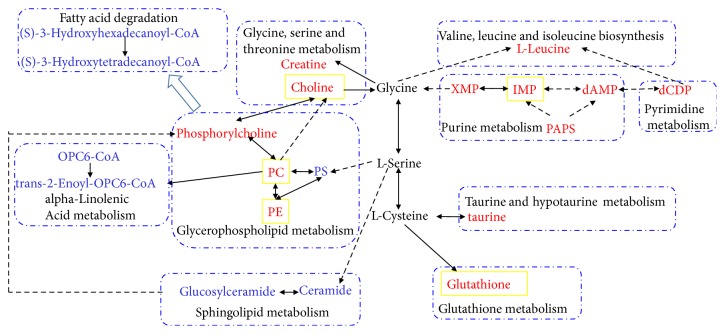
Schematic diagram of the main related metabolic pathways related to immunoregulation of PG. PC: PC (18:3(9Z,12Z,15Z)/15:0), PE:PE (18:0/16:1(9Z)), and PS:PS (18:0/20:4(8Z,11Z,14Z,17Z)). The red and light blue words indicate metabolites significantly increased and reduced, respectively, in the PG-treated group compared with the control group. The yellow boxes indicate metabolites that are affected by PG using network pharmacology analysis. The solid lines indicate that one metabolite produces another metabolite in less than two steps in KEGG metabolism pathway maps.

In this study, the effectiveness of PG on enhancing macrophage phagocytosis of GFP-*E. coli* was systematically analyzed by LC-MS-based metabolomics and network pharmacology. Based on the metabolomics analysis, obvious metabolic alteration of lipids metabolism, nucleotide metabolism, and amino acids metabolism was found in the PG-treated samples. Lipid metabolisms are always activated by inflammatory mediators [33]. Moreover, lipid-mediated signaling is also intimately involved in phagosome formation and maturation [34]. As shown in Figure 7, the identified metabolites of glycerophospholipids metabolism, sphingolipids metabolism, and fatty acid degradation decreased in the PG-treated group in comparison to control group. The changes of glycerophospholipid have been reported as an initial event of phagocytosis and fatty acid has effects on macrophage phagocytosis [35]. On the other hand, sphingolipid metabolism is closely related to some immune cytokines (such as TNF-*α*) and can further promote phagolysosome formation [36]. On the contrary, glutathione, taurine, isoleucine, creatine, phosphorylcholine, and choline were all increased in PG-treated group in comparison to control group, which are the metabolites of amino acids metabolism. The changes of those metabolites may reflect the enhancement of amino acids metabolism. It has also been proved that the cell phagocytic ability could be enhanced through the increased turnover of glutathione [37], creatine [38], or taurine [39], as well as the activity of some leucine metabolic enzymes [40]. Phosphorylcholine and choline are the hydrolysates of PC and can be accumulated by activating cytoplasmic phospholipase A2 through macrophage colony stimulating factor [41].

Furthermore, about 20 compounds in PG may contribute to the enhancement of macrophage phagocytosis according to the analysis of network pharmacology, including ginsenosides, phytosterols, sesquiterpenes, flavonoids, and alkaloids. Ginsenosides are often considered as the major active component of PG and our results also indicated that 6 ginsenosides (ginsenoside Rb2, ginsenoside Re, ginsenoside Rc, ginsenoside Rg1, ginsenoside Rg2, and Ginsenoside Rb1) may activate the macrophage phagocytosis by regulating glutathione metabolism through glutathione S-transferase P or glutathione S-transferase Mu 1. It has been reported that the glutathione-S-transferase–PAP (phagocytosis activating protein) can increase percentage of haemocyte phagocytosis of shrimps [42]. Besides, some other components of PG also show their effects on macrophage phagocytosis in this study. For example, kaempferol, frutinone A, humulene oxide, vulgarin, 3-Ethyl-3-methylheptane, neohexane, mannose-b, 3-methylheptane, and MAV were found to have potential effect on regulating the choline metabolism through acetylcholinesterase or cholinesterase. Kaempferol and ginsenosides also show synergistic effects on regulating the glutathione metabolism. Among these ingredients of PG, ginsenoside Re [43], ginsenoside Rg1 [44], ginsenoside Rb1 [45], kaempferol [46], and mannose-b [47] reportedly enhance the phagocytosis effect or immune responses. In summary, the compounds of PG enhance the macrophage phagocytosis mainly by regulating glutathione metabolism, glycerophospholipid metabolism, and choline metabolism according to the comprehensively network pharmacology analysis. Among these compounds, ginsenosides mainly affect glutathione metabolism through glutathione S-transferase. Other compounds can regulate the choline metabolism and glycerophospholipid metabolism. To further illustrate the pharmacodynamic action of PG, the potential targets predicted by network pharmacology were classified and analyzed with their functions in M1 and M2 macrophages. The results showed that PG can activate both M1 and M2 macrophage but may be prone to activate M2 macrophages, which possess immune activation feature for host defense with phagocytic capacity and bacterial clearance ability [48]. Ginsenosides Rb1 could polarize M1 macrophages to M2 macrophages when increasing the phagocytic ability of macrophages toward* E. coli *[45].

In conclusion, this study provided a holistic view in the molecular mechanism of PG enhancing the macrophage phagocytosis with the combination of metabolomics and network pharmacology analyses. Our results also indicated that PG may primarily skew the macrophages to the M2 phenotype by regulating the glutathione and choline metabolism. These findings will help us to take more insight into the immunoregulatory effects of PG. Further studies will evaluate and validate the efficiency of the main targets and constituents of PG in our study.

## Figures and Tables

**Table 1:** Identification results of potential biomarkers.

tR(min)	Metabolites	Mass (Neutral)	Error (ppm)	Formulate	VIP	Fold	p^a^
Data from the ESI- mode							
0.94	OPC6-CoA	1015.2662	26.23677	C_37_H_60_N_7_O_18_P_3_S	1.20	1.34	0.016
0.96	trans-2-Enoyl-OPC6-CoA	1013.2888	-11.4598	C_37_H_58_N_7_O_18_P_3_S	1.20	1.54	0.018
0.97	PC (18:3(9Z,12Z,15Z)/15:0)	741.5355	-6.25561	C_41_H_77_NO_8_P	1.36	1.59	0.004
0.99	PS (18:0/20:4(8Z,11Z,14Z,17Z))	811.5429	-8.09083	C_44_H_78_NO_10_P	1.37	1.57	0.016
1.04	Phosphorylcholine	169.0488	9.373073	C_4_H_13_NO_4_P	1.16	0.58	0.011
0.96	PE (18:0/16:1(9Z))	717.5429	-16.7632	C_39_H_77_NO_8_P	1.17	1.56	0.005
0.97	Glucosylceramide (d18:1/16:0)	699.5484	23.61182	C_40_H_77_NO_8_	1.03	1.36	0.004
0.93	Ceramide (d18:1/20:0)	593.5822	-12.6437	C_38_H_75_NO_3_	1.34	1.83	0.002
0.97	2′-Deoxycytidine diphosphate (dCDP)	387.0237	-1.1188	C_9_H_15_N_3_O_10_P_2_	1.24	0.64	0.008
0.99	Xanthylic acid (XMP)	364.042	0.041204	C_10_H_13_N_4_O_9_P	1.17	0.56	0.016
0.96	Deoxyadenosine monophosphate (dAMP)	331.0645	11.08533	C_10_H_14_N_5_O_6_P	1.20	0.67	0.012
0.99	3′-Phosphoadenylyl sulfate (PAPS)	506.9801	12.08909	C_10_H_15_N_5_O_13_P_2_S	1.22	0.64	0.001
0.96	Glutathione	307.0832	1.973403	C_10_H_17_N_3_O_6_S	1.19	0.61	0.014
1.03	Taurine	125.015	-2.68768	C_2_H_7_NO_3_S	1.33	0.54	0.004
1	Inosine 5′-monophosphate (IMP)	348.0491	-5.7466	C_10_H_13_N_4_O_8_P	1.04	0.35	0.002
0.97	(S)-3-Hydroxytetradecanoyl-CoA	993.335	-26.6906	C_35_H_62_N_7_O_18_P_3_S	1.27	1.58	0.007
0.97	(S)-3-Hydroxyhexadecanoyl-CoA	1021.3628	-22.5302	C_37_H_66_N_7_O_18_P_3_S	1.21	1.56	0.002
Data from the ESI+ mode							
1.02	Choline	103.0996	1.094715	C_5_H_14_NO	1.31	0.60	0.026
1.07	Creatine	131.0686	6.691108	C_4_H_9_N_3_O_2_	0.99	0.65	0.047
1.07	L-Isoleucine	131.0919	20.81702	C_6_H_13_NO_2_	0.76	0.66	0.025

**Table 2:** The KEGG and GO pathway analysis.

Term	P value	Benjamini	Related proteins
KEGG Term			
Glutathione metabolism	0.00079	0.025	P09211, P28161, P09488
Drug metabolism - cytochrome P450	0.0014	0.022	P09211, P28161, P09488
Metabolism of xenobiotics by cytochrome P450	0.0017	0.018	P09211, P28161, P09488
Chemical carcinogenesis	0.0019	0.015	P09211, P28161, P09488
Glycerophospholipid metabolism	0.08	0.41	P22303, P47712
Go Term (Biological Process)			
glutathione derivative biosynthetic process	0.000034	0.0039	P09211, P28161, P09488
glutathione metabolic process	0.00023	0.013	P09211, P28161, P09488
cellular detoxification of nitrogen compound	0.0013	0.046	P28161, P09488
nitrobenzene metabolic process	0.0017	0.046	P28161, P09488
metabolic process	0.002	0.045	P09211, P28161, P09488
xenobiotic catabolic process	0.0029	0.053	P28161, P09488
linoleic acid metabolic process	0.0071	0.11	P09211, P28161
cellular oxidant detoxification	0.029	0.34	P09211, P28161
Go Term (Cellular Component)			
cytosol	0.003	0.086	P11217, P09211, P28161, P30520, P09488, P47712
cytoplasm	0.024	0.3	P11217, P09211, P28161, P30520, P09488, P47712
extracellular exosome	0.079	0.56	P11217, P09211, P28161, P30520
Go Term (molecular function)			
glutathione binding	8.1E-06	0.00033	P09211, P28161, P09488
glutathione transferase activity	0.000087	0.0018	P09211, P28161, P09488
acetylcholinesterase activity	0.00083	0.011	P22303, P06276
cholinesterase activity	0.0012	0.013	P22303, P06276
enzyme binding	0.0076	0.061	P28161, P09488, P06276
glutathione peroxidase activity	0.0087	0.058	P09211, P28161
beta-amyloid binding	0.014	0.079	P22303, P06276
protein homodimerization activity	0.034	0.16	P28161, P09488, P22303
transferase activity	0.039	0.17	P09211, P09488

## Data Availability

The data used to support the findings of this study are available from the corresponding author upon request.

## Supplementary Materials

Supplementary Materials Figure S1 shows the study on methodology of phagocytosis activity. Figure S2 shows the UPLC profile of PG extract and the reference mixture. Figure S3 shows macrophage survival rate of cotreatment with PG extracts and the LPS, detected by CCK8 method. Table S1 shows the information of potential biomarkers. Table S2 shows parameters of PCA and OPLS-DA models of the metabolomics results. Table S3 shows the results from pathway analysis of potential biomarkers with MetaboAnalyst 3.0. Table S4 shows the ADME indexes of PG's ingredients by network pharmacology analyses. Table S5 shows the details of metabolic proteins through network pharmacology analyses. Table S6 shows proteins in M1 and M2 macrophages associated with potential biomarkers of PG.
