# Supplementary material for: Integrated Metabolomics and Network Pharmacology Study on Immunoregulation Mechanisms of Panax ginseng through Macrophages
Source: Evid Based Complement Alternat Med. 2019 Jun 25;2019:3630260. doi: 10.1155/2019/3630260 (PMC6614982; doi:10.1155/2019/3630260)
Supplement: Supplementary Materials — Figure S1 shows the study on methodology of phagocytosis activity. Figure S2 shows the UPLC profile of PG extract and the reference mixture. Figure S3 shows macrophage survival rate of cotreatment with PG extracts and the LPS, detected by CCK8 method. Table S1 shows the information of potential biomarkers. Table S2 shows parameters of PCA and OPLS-DA models of the metabolomics results. Table S3 shows the results from pathway analysis of potential biomarkers with MetaboAnalyst 3.0. Table S4 shows the ADME indexes of PG's ingredients by network pharmacology analyses. Table S5 shows the details of metabolic proteins through network pharmacology analyses. Table S6 shows proteins in M1 and M2 macrophages associated with potential biomarkers of PG. [file 3630260.f1.pdf]

Supplementary Materials:

The influence of different incubation time (0.5, 1, 2, 3 h) was studied when the GFP-*E. coli* number/cell number was 50. The other conditions are the same as the content of **2.4 High content screening analyses of phagocytosis activity**. The average phagocytic index sustained increased in 0.5-2 h incubating but decreased at 3h, so we incubated for 2 h (Figure 1 A). Then we study influence of different GFP-*E. coli* number (GFP-*E. coli* number/cell number was from 12.5 to 500). As shown in Figure S1 B and C, The average phagocytic index increased when the GFP-*E. coli* number/cell number was from 12.5 to 400, while the cell number decreased when GFP-*E. coli* number/cell number was 200. Therefore, the GFP-*E. coli* number/cell number was determined to be 100 and incubation time was 2 h.

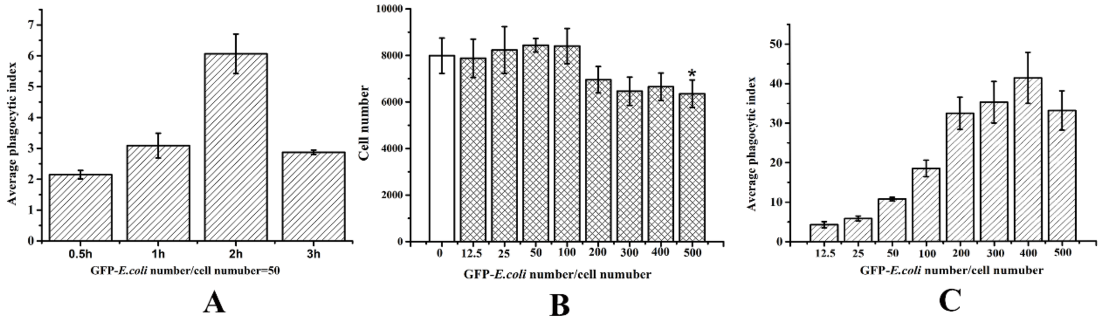

FIGURE S1: The study on methodology of phagocytosis activity. (A) The average phagocytic index of macrophage in different time incubating with GFP-*E. coli*. (B, C) The cell number and average phagocytic index when the GFP-*E. coli* number/cell number was from 12.5 to 400. *P<0.05 vs the group without GFP-*E. coli*.

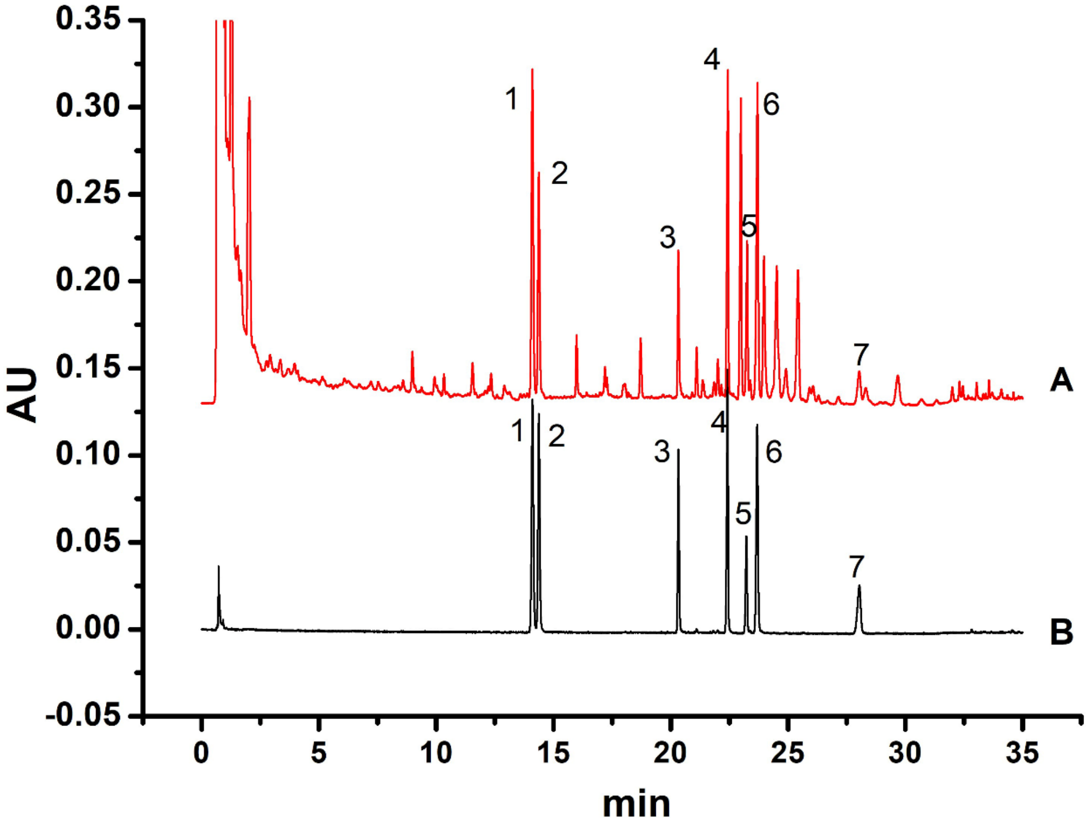

FIGURE S2: UPLC ginsenoside profile of PG: (A) PG extract; (B) Standards. Detection at 203 nm wavelength. Peaks: (1) ginsenoside Rg1; (2) ginsenoside Re; (3) ginsenoside Rf; (4) ginsenosideRb1; (5) ginsenoside Rc; (6) ginsenoside Ro; (7) ginsenoside Rd.

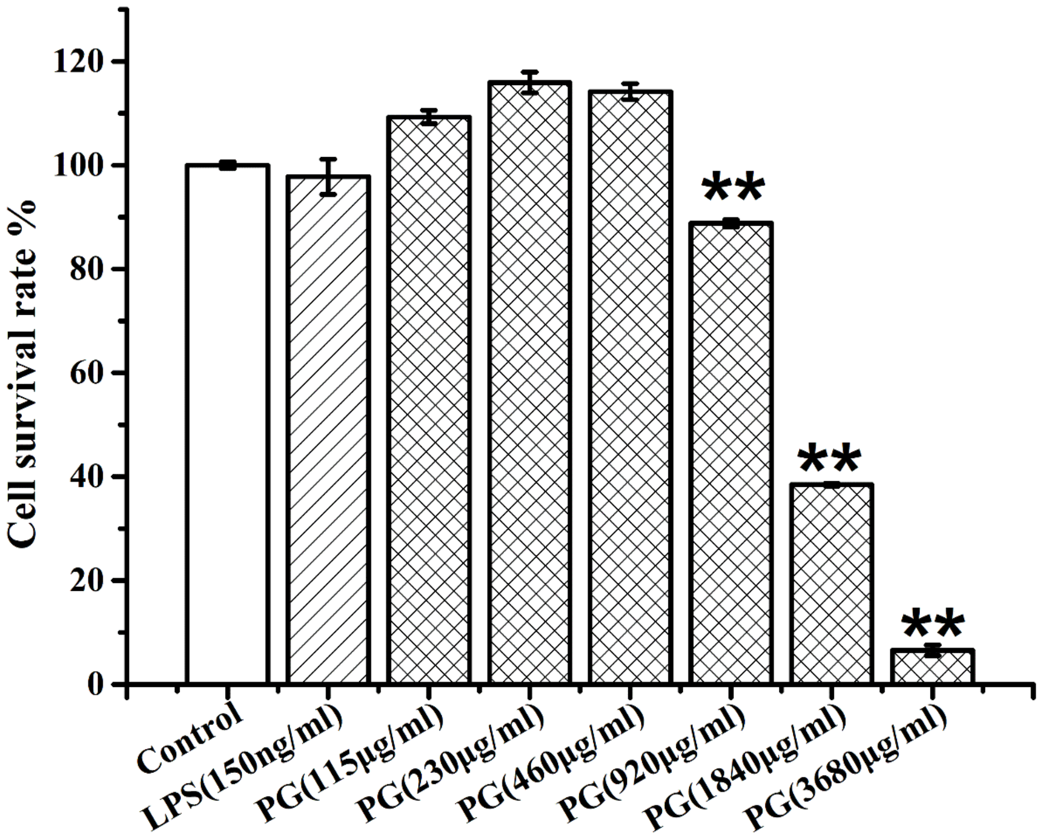

FIGURE S3: Macrophage survival rate of co-treatment with PG extracts (115-3680 μg/ml) and the LPS (150 ng/ml), detected by CCK8 method. ***P*<0.01 vs control group.

TABLE S1: The information of potential biomarkers.

Metabolites	KEGG ID	Pathway
Data from the ESI- mode		
OPC6-CoA	C16331	alpha-Linolenic acid metabolism
trans-2-Enoyl-OPC6-CoA	C16332	alpha-Linolenic acid metabolism
PC(18:3(9Z,12Z,15Z)/15:0)	C00157	Glycerophospholipid metabolism
PS(18:0/20:4(8Z,11Z,14Z,17Z))	C02737	Glycerophospholipid metabolism
Phosphorylcholine	C00588	Glycerophospholipid metabolism
PE(18:0/16:1(9Z))	C00350	Glycerophospholipid metabolism
Glucosylceramide (d18:1/16:0)	C01190	Sphingolipid metabolism
Ceramide (d18:1/20:0)	C00195	Sphingolipid metabolism
2'-Deoxycytidine diphosphate (dCDP)	C00705	Pyrimidine metabolism
Xanthylic acid（XMP）	C00655	Purine metabolism
Deoxyadenosine monophosphate （dAMP）	C00360	Purine metabolism
3'-Phosphoadenylyl sulfate (PAPS)	C00053	Purine metabolism
Glutathione	C00051	Glutathione metabolism
Taurine	C00245	Taurine and hypotaurine metabolism
Inosine 5'-monophosphate (IMP)	C00130	Purine metabolism
(S)-3-Hydroxytetradecanoyl-CoA	C05260	Fatty acid degradation
(S)-3-Hydroxyhexadecanoyl-CoA	C05258	Fatty acid degradation
Data from the ESI+ mode		
Choline	C00114	Glycine, serine and threonine metabolism
Creatine	C00300	Glycine, serine and threonine metabolism
L-IsoLeucine	C00123	Valine, leucine and isoleucine biosynthesis

TABLE S2: Parameters of PCA and OPLS-DA models.

Model No.	Model type	Groups included	Component	R2X	R2Y (cum)	Q2Y (cum)
Data of ESI- mode						
M1	PCA	-	2	0.657	-	0.455
M2	OPLS-DA	PG-High & Control	1+1+0	0.837	0.999	0.985
Data of ESI+ mode						
M3	PCA	-	3	0.641	-	0.153
M4	OPLS-DA	PG-High & Control	1+3+0	0.891	1	0.982

TABLE S3: Results from pathway analysis of potential biomarkers with MetaboAnalyst 3.0.

Pathway name	Total	Hits	RAW p	FDR	Impact
Glycerophospholipid metabolism	30	5	4.83E-05	0.003964	0.32284
alpha-Linolenic acid metabolism	9	3	0.000223	0.009139	0
Purine metabolism	68	4	0.015644	0.4276	0.08939
Sphingolipid metabolism	21	2	0.03709	0.76034	0.32331
Fatty acid elongation in mitochondria	27	2	0.058781	0.87982	0.08606
Sulfur metabolism	5	1	0.072035	0.87982	0.3
Glycine, serine and threonine metabolism	31	2	0.075169	0.87982	0
Linoleic acid metabolism	6	1	0.085836	0.87982	0
Fatty acid metabolism	39	2	0.11165	0.9254	0.08739
Taurine and hypotaurine metabolism	8	1	0.11285	0.9254	0.42857
Valine, leucine and isoleucine biosynthesis	11	1	0.15196	1	0.33333
Glycosylphosphatidylinositol (GPI)-anchor biosynthesis	14	1	0.18942	1	0.0439
Glutathione metabolism	26	1	0.32409	1	0.36069
Arachidonic acid metabolism	36	1	0.41976	1	0
Valine, leucine and isoleucine degradation	38	1	0.43728	1	0
Pyrimidine metabolism	41	1	0.46262	1	0.0364
Arginine and proline metabolism	44	1	0.48687	1	0.01198
Primary bile acid biosynthesis	46	1	0.50245	1	0.02976
Aminoacyl-tRNA biosynthesis	69	1	0.65215	1	0
Steroid hormone biosynthesis	72	1	0.66817	1	0.02711

TABLE S4: The ADME indexes of PG's ingredients by network pharmacology analyses.

Molecule name	OB (%)	Caco-2	DL
Malonic acid	21.69	-0.23	0.01
Tridecanoic acid	22.32	1.04	0.05
Stigmasterol	43.83	1.44	0.76
beta-Elemene	25.63	1.84	0.06
3-Ethyl-3-methylheptane	37.33	1.81	0.02
beta-Sitosterol	36.91	1.32	0.75
Neohexane	37.81	1.77	0.01
Frutinone A	65.9	0.89	0.34
Humulene oxide	23.66	1.58	0.1
Kaempferol	41.88	0.26	0.24
Vulgarin	29.21	0.03	0.2
Mannose-b	43.04	-1.82	0.04
3-methylheptane	37.1	1.8	0.01
(2S,3S,4S,5S,6S)-3,4,5,6-tetrahydroxyoxane-2-carboxylic acid(MAV)	56.17	-2.14	0.06
Ginsenoside Rb2	6.02	-3.92	0.04
Ginsenoside Re	4.27	-3.2	0.12
Ginsenoside Rc	8.16	-3.97	0.04
Ginsenoside Rg1	10.2	0.12	0.78
Ginsenoside Rg2	20.12	-0.04	0.82
Ginsenoside Rb1	6.24	-3.99	0.04

TABLE S5: The details of metabolic proteins through network pharmacology analyses.

UniProt ID	Protein name
P47712	Cytosolic phospholipase A2
Q15596	Nuclear receptor coactivator 2
P38936	Cyclin-dependent kinase inhibitor 1
P28161	Glutathione S-transferase Mu 2
P09211	Glutathione S-transferase P
P09488	Glutathione S-transferase Mu 1
P22303	Acetylcholinesterase
P06276	Cholinesterase
P11217	Glycogen phosphorylase, muscle form
P30520	Adenylosuccinate synthetase isozyme 2
Q9Y6K0	Choline/ethanolaminephosphotransferase 1

TABLE S6: Proteins in M1 and M2 macrophage associated with potential biomarkers of PG.

Protein ID	Gene Symbol	Description
	M1	
P37238	Pparg	Peroxisome proliferator activated receptor gamma
	M2	
P58681	Tlr7	Toll like receptor 7
Q61503	Nt5e	5'-Nucleotidase ecto
P70206	Plxna1	Plexin A1
P12025	Mdk	Midkine
P70207	Plxna2	Plexin A2
P07141	Csf1	Colony stimulating factor 1
Q62151	Ager	Advanced glycosylation end-product specific receptor
O88786	Il13ra2	Interleukin 13 receptor subunit alpha 2
P20109	Il13	Interleukin 13
P07214	Sparc	Secreted protein acidic and cysteine rich
P14901	Hmox1	Heme oxygenase 1
P16045	Lgals1	Galectin 1
P52633	Stat6	Signal transducer and activator of transcription 6
P13504	Il1r1	Interleukin 1 receptor type 1
P09803	Cdh1	Cadherin 1
P11835	Itgb2	Integrin subunit beta 2
P21981	Tgm2	Transglutaminase 2
ENSMUSG00000055170	Ifng	Interferon gamma
Q9WVH4	Foxo3	Forkhead box o3
P42227	Stat3	Signal transducer and activator of transcription 3
P42225	Stat1	Signal transducer and activator of transcription 1
P25799	Nfkb1	nuclear factor kappa B subunit 1
P11276	Fn1	Fibronectin 1
P35441	Thbs1	Thrombospondin 1
P26231	Ctnna1	Catenin alpha 1
Q61362	Chi3l1	Chitinase 3 like 1
ENSMUSG00000002603	Tgfb1	Transforming growth factor beta 1
Q02248	Ctnnb1	Catenin beta 1
Q9R1E0	Foxo1	Forkhead box o1
Q61221	Hif1a	Hypoxia inducible factor 1 subunit alpha
	Both M1 and M2	
ENSMUSG00000000869	Il4	Interleukin 4
Q61176	Arg1	Arginase 1
ENSMUSG00000025746	Il6	Interleukin 6
P31041	Cd28	CD28 molecule

Q8K4B2	Irak3	Interleukin 1 receptor associated kinase 3
P06804	Tnf	Tumor necrosis factor
P29477	Nos2	Nitric oxide synthase 2
